# Social Support, Stigma and Disclosure: Examining the Relationship with HIV Medication Adherence among Ryan White Program Clients in the Mid-South USA

**DOI:** 10.3390/ijerph120607073

**Published:** 2015-06-19

**Authors:** Latrice C. Pichon, Kristen R. Rossi, Siri A. Ogg, Lisa J. Krull, Dorcas Young Griffin

**Affiliations:** 1 School of Public Health, University of Memphis, Robison Hall, Memphis, TN 38152, USA; E-Mails: saogg@memphis.edu (S.A.O.); ljkrull@memphis.edu (L.J.K.); 2 Shelby County Health Department Epidemiology Section, 814 Jefferson Ave., Memphis, TN 38105, USA; E-Mail: kristenraerossi@gmail.com; 3 Shelby County Government, 160 N. Main St., Suite 250, Memphis, TN 38103 USA; E-Mail: Dorcas.Young@shelbycountytn.gov

**Keywords:** HIV, medication adherence, patient care, CBPR, church

## Abstract

Social support from friends and family is positively related to better health outcomes among adults living with HIV. An extension of these networks such as religious communities may be an untapped source of social support for promoting HIV medical adherence. This paper explores the association of HIV medication adherence to satisfaction with support from family, friends and church members, as well as HIV-related stigma, and HIV disclosure. In partnership with the Shelby County Health Department, the Memphis Ryan White Part A Program, and the University of Memphis School of Public Health, a total of 286 interviewer-administered surveys were conducted with Ryan White clients. Seventy-six percent (*n* = 216) of participants reported being prescribed antiretroviral medication (ARVs). Nearly all participants (*n* = 202, 94%) prescribed ARVs reported disclosing their HIV status to someone. Almost 20% (*n* = 40) of those prescribed ARVs reported not being satisfied with support received from his/her church. Interestingly, participants reported rarely experiencing stigma as a result of their HIV status. The extent to which satisfaction with support from personal networks and institutional settings like the church affect medication adherence is yet to be understood. The complexity of HIV disclosure and HIV stigma in relation to these supports warrants further investigation to understand how best to improve HIV health outcomes.

## 1. Introduction

Anti-Retroviral therapy has been proven effective in reducing mortality from HIV, transforming the disease into a manageable chronic illness rather than a terminal diagnosis [1] However, the treatment is only effective with a strict level of medical adherence. While increased knowledge about the disease, positive expectations of treatment efficacy, and less complicated drug regimens facilitate adherence [2], medication side effects, life responsibilities and stressors, and depression may diminish compliance [3]. Although the role of social support is complicated and not fully understood in this context [4], it may also affect adherence.

Social support is positively related to better health outcomes in several disease contexts. However, numerous types of support, their sources and pathways, make it difficult to pinpoint how this process is fully manifested, especially in relation to HIV [5]. For instance, perceived *vs.* actual support and emotional *vs.* instrumental support fulfill different needs in each person’s life [4]. Social support comes in many forms and from various parties [6]. Much of the literature on social support and HIV outcomes, including medication adherence, focuses on the support provided by family and partners, but it may also be important to consider an expanded social support network, including that provided by a church.

For U.S. Blacks infected with HIV and living in the South, faith communities may be an untapped source of social support for promoting HIV medical adherence. Research demonstrates that 85% of Blacks report religion as being very important to them, and more than half of Blacks report attending religious services at least once a week (PEW, 2012). Moreover, 64% of Blacks living in the South are members of Historically Black Churches. Thus, faith, religion, spirituality, and the church play a critical role in the social fabric of the Black community, especially in the South. Members of a church family may also provide the same instrumental and emotional support as relatives, friends and partners [7]. However, social support from the church context may involve HIV-related stigma; in spite of increased awareness and education about HIV in faith contexts, dynamics such as blaming the victim and unfounded fears of transmission from casual contact remain [3]. In fact, stigma may keep HIV-positive individuals from disclosing their status to family and friends, as well as to church members, preventing them from receiving support that may be beneficial and helpful. Many who disclose their status have experienced adverse reactions, negatively affecting their self-esteem and future reliance on others for support [6]. While HIV-related stigma and disclosure must be considered, the potential positive role of social support on HIV health outcomes cannot be ignored.

In current practice, this role has been explored by a local government program administering Ryan White Part A funds to provide medical and supportive services for persons living with HIV (PLWH) in the Memphis Metropolitan Statistical Area (MSA). In the Memphis MSA, 43% of PLWH (3378 of 7856) had no documented medical care for their HIV disease during 2011. In response, the Memphis Ryan White Program began recruiting faith leaders in an effort to raise awareness about the disease in the community, and to develop additional avenues for linking infected individuals to medical care. The role of the faith community with regards to support for HIV care was further assessed within a structured needs assessment. This paper aims to examine the association of HIV medication adherence to satisfaction with support provided by various social network members, including family, friends, and church members, as well as HIV-related stigma and HIV disclosure.

## 2. Methods

### 2.1. Design

The Memphis Transitional Grant Area (TGA) Ryan White Program provides medical and supportive services for PLWH at or below 300% of Federal Poverty Level (FPL), and residing in one of the eight counties within the MSA, which encompasses three states. As required by the Ryan White CARE Act legislation, all Part A grantees conduct periodic Needs Assessments to provide data-driven recommendations for priority setting and resource allocation of funds [8]. A mixed-methods approach, using community-based participatory research [9,10], was employed to conduct the 2012 Needs Assessment, which aimed to determine service gaps, barriers and needs in the continuum of care for PLWH. This paper will focus exclusively on the quantitative analysis.

The overall Needs Assessment objectives were guided by the Behavioral Model for Vulnerable Populations, which describes factors that may enable or impede health services utilization and access to care, including those at the individual, interpersonal, and community-levels [11]. Key results from quantitative surveys are described in this paper to evaluate satisfaction with social support from family, friends, and the church, HIV-related stigma, and HIV disclosure on medication adherence. This study was approved by the University of Memphis (110711-974), St. Jude Children’s Research Hospital, The Regional Medical Center, and the University of Tennessee Health Sciences Research Ethics Committees (St Jude IRB XPD12-014). 

### 2.2. Eligibility and Recruitment

All PLWH, aged 18 and older, and eligible for Ryan White Part A services in the Memphis MSA were recruited for participation. Medical service providers familiar with the participants (e.g., case managers) and university-based research staff conducted recruitment for the quantitative survey on-site Ryan White-funded service provider locations. University-based research staff also administered surveys with clients attending various public meetings held by the Ryan White program. Survey respondents were provided a $10 grocery gift card to compensate their time.

### 2.3. Survey

A total of 286 quantitative surveys were conducted among a convenience-based sample of clients meeting eligibility requirements. We included previously used survey items from other needs assessments conducted nationally and by our local community. A panel of community experts, including clients utilizing Ryan White services, reviewed the survey instrument to assess readability, ease of administration, and comprehension prior to implementation. We added existing measures from the literature for domains identified to be missing in previous assessments based on the consensus of this panel. Descriptive information such as demographic factors including, age, race, and sexual identity were collected.

Outcomes related to medication adherence were assessed through a panel of survey items. First, participants where asked whether or not they had received antiretroviral medication (ARVs) within the past 12 months. Among those who did receive ARVs, adherence was assessed over the past 30 days and last week (seven days); participants were asked if they had taken all, most, half, few, or none of their antiretroviral medication. Among participants who had less than optimal adherence during the past 30 days (taken most, half, few, or none of their medication), a follow-up question was posed to document the reasons why the individual missed taking any dose. Responses were categorized into pre-determined check boxes, but an open-ended “other” response was available to add free text as necessary.

Satisfaction with support received from (1) friends/family; and (2) church members was assessed separately; participants indicated if they were very satisfied, somewhat satisfied, somewhat dissatisfied or very dissatisfied regarding the support they receive from (1) family/friends; and (2) members of their church. The relationship between medication adherence and overall satisfaction with social support was further assessed with two additional survey items; (1) participants were asked whether or not family members helped them remember to take their medication (yes/no); and (2) they were also asked to describe the extent members of their church helped them remember to take their medication (not at all, a little, a lot, or not applicable). To assess HIV disclosure, participants were asked if they had disclosed their HIV status to anyone (yes/no); if yes, a follow-up item asked to whom they had disclosed their positive status (e.g., sexual partners, friends, siblings, and parents). Lastly, participants were asked how often they had experienced HIV-related stigma on a scale of one to four, where one signified often and four signified never.

### 2.4. Analysis

Survey data were entered into and analyzed by the statistical Software Program for the Social Sciences (SPSS) Statistics 21 (IBM, Chicago, IL, USA). Of the 286 surveys collected, 216 clients reported being prescribed ARVs. Thus, the analysis was restricted to these clients and demographic data is reported for them only. Chi-square analyses (χ^2^) and independent *t*-tests were conducted to evaluate significant differences between potential correlates (e.g., HIV disclosure and HIV-related stigma) with outcomes related to medication adherence (optimal adherence *vs.* missing at least one dose), as well as significant bivariate associations between satisfaction with social support (from friends/family and church members) and medication adherence.

## 3. Results

### 3.1. Participant Characteristics

Of the 216 participants indicating they had received ARVs within the past 12 months, the age distribution ranged from 18 to 66 years, with an average age of 40 years. Over two-thirds of the clients were male; 88% self-identified as non-Hispanic Black or African American. Participant characteristics are reported in Table 1 (note: characteristics for non-participant clients receiving services from the Ryan White program were similar to participants).

**Table 1:** Demographic Characteristics for Respondents who Received ARVs within the Past 12 Months (*n* = 216).

Demographic Characteristics	*n*	%
**Sexual Identity**		
Male	145	67.1
Female	70	32.4
Transgender	1	0.5
**Age**		
18–24	31	14.4
25–34	37	17.1
35–44	54	25
45–54	70	32.4
55+	20	9.3
**Education**		
<High School	48	22.2
High School Graduate/GED	75	34.7
Some College	71	32.9
College Graduate	16	7.4
Graduate Degree	4	1.9
**Employment**		
Full-time ≥40 h	22	10.2
Part-time <40 h	19	8.8
Unemployed	68	31.5
Disability	78	36.1
Other (e.g., retired, student)	28	13.0
**Race**		
Black	191	88.4
White	14	6.5
Other	11	5.1
Single	143	66.2
**Relationship Status**		
Married/Living w/Partner	34	15.7
Steady Partner (not living together)	21	9.7
Separated/Divorced/Widowed	18	8.3

Missing data for age (*n* = 4) and employment (*n* = 1); two participants reported “other” for education.

### 3.2. Survey Findings

#### 3.2.1. Medication Adherence

Among the 286 survey respondents, approximately 76% (*n* = 216) received ARVs in the last 12 months. Of those, 151 participants (69.9%) missed taking their medication within the past month. For those who reported missing doses, the most common reasons ranged from forgetting (*n* = 82, 54.3%), being away from home (*n* = 58, 38.4%), having a change in daily routine (*n* = 48, 31.7%), and running out of pills (*n* = 44, 29.1%). Survey participants also were asked about medication adherence over the last seven days. Among those missing a dose within the past month, approximately 29% self-reported less than optimal adherence levels (*n* = 44 missed at least one dose) over the last seven days (Table 2).

**Table 2:** Medication Adherence among Respondents who Received ARVs within the Past 12 Months (*n* = 216).

Adherence Issue	*n*	%
Missed a dose within the past month	151	69.9
Within the past 7 days *	44	29.1
Reasons for missing a dose in the past 12 months		
Forgetting	82	54.3
Being away from home	58	38.4
Change in daily routine	48	31.7
Running out of pills	44	29.1

* Percentage calculated among those who missed a dose within the past month.

#### 3.2.2. Social Support and Disclosure

Nearly all participants (*n* = 202, 94%) prescribed ARVs reported disclosing their HIV status to someone. The most common individuals to whom HIV status was disclosed included sexual partners (79.2%), siblings (70.4%), friends (69.9%) and parents (69.4%). Participants were asked about the level of satisfaction with regard to support from family/friends, and the church members. Approximately 36% (*n* = 78) of respondents who reported taking ARVs were less than *very satisfied* with support received from their friends/family members. Additionally, 18.5% (*n* = 40) reported being less than *very satisfied* with support received from his or her church members. Approximately 53% (*n* = 114) of participants responded *no* to friends/family helped them to remember to take their medication, and 19% (*n* = 42) stated the church members were *not at all* helpful with regard to medication adherence (Table 3).

Upon examining the relationship between social support with adherence, approximately 43% (*n* = 74) of those who received support/reminders from friends/family for medication took all of their doses over a seven day period compared to 57% (*n* = 97) who did not receive this support (χ^2^ = 4.597, *p*-value = 0.032). There were no other significant measures of social support or HIV disclosure with adherence (data not shown). 

#### 3.2.3. Stigma

Interestingly, respondents more commonly reported *never* or *rarely* experiencing HIV-related stigma as opposed to *sometimes* or *often*, as shown in Table 4. The most frequently cited HIV-stigma scale questions respondents *sometimes* or *often* felt included: “Thought other people were uncomfortable being with you” (43.1%), “feared you would lose friends if they learned of your diagnosis” (39.8%), and “thought your diagnosis was punishment for things done in the past” (38.5%). There were no significant associations between HIV-related stigma and medication adherence.

**Table 3:** HIV Disclosure and Perceived Social Support among Respondents who Received ARVs within the Past 12 Months (*n* = 216).

Disclosure and Social Support		*n*		%	
**HIV Disclosure**					
Sexual Partners		171		79.2	
Siblings		152		70.4	
Friends		151		69.9	
Parents		150		69.4	
Children		74		34.3	
Employer		45		20.8	
**Perceived Support from Family and/or Friends**					
Very Satisfied		123		56.9	
Somewhat satisfied		54		25.0	
Somewhat dissatisfied		14		6.5	
Very dissatisfied		10		4.6	
**Perceived Support from Church**					
Very Satisfied		66		30.6	
Somewhat satisfied		25		11.6	
Somewhat dissatisfied		5		2.3	
Very dissatisfied		10		4.6	
**Medication Reminders**					
From family or friends					
Yes		102		47.2	
From church members					
Not at all		42		19.4	
A little		12		5.6	
A lot		16		7.4	
N/A		145		67.1	

**Table 4:** HIV Stigma Scale Responses among Respondents Who Received ARVs within the Past 12 Months (*n* = 216).

HIV Stigma	Rarely/Never		Sometimes/Often	
	Felt This Way		Felt This Way	
	*n*	%	*n*	%
Thought other people were uncomfortable being with you	117	54.1	93	43.1
Feared you would lose friends if they learned about diagnosis	123	56.9	86	39.8
Thought your diagnosis was punishment for things done in the past	128	59.2	83	38.5
Feared losing job if someone found out	126	58.3	77	35.7
Felt people avoiding you because of diagnosis	134	62.1	76	35.1
Feared family would reject you if they learned about diagnosis	141	65.3	67	31.0
Felt blamed by others for diagnosis	154	71.2	58	26.9
Felt compelled to change residence because of diagnosis	160	74.1	49	22.7
Felt you wouldn’t get as good health care if people learned about diagnosis	171	79.2	39	18.0
Avoided getting treatment because someone might find out	178	82.4	31	14.3
Feared people might hurt your family if they learned of your diagnosis	181	83.8	29	13.4

## 4. Discussion

This analysis sought to explore associations of medication adherence to satisfaction with social support, HIV-related stigma, and HIV disclosure among a convenience-based sample of adults accessing HIV medical and support services. We found relatively high non-adherence rates, where almost three-quarters self-reported missing medication doses during last month. This is particularly alarming as the participants recruited for this study were currently engaged in HIV care at the time of the survey.

Sources of support in our study included individuals with both formal and informal roles (e.g., medical and other care providers, family, and friends) and institutions, (e.g., churches) which is congruent with other literature [12]. However, our findings diverge from the findings of one study demonstrating that having support from family and friends was associated with encouraging medication adherence, keeping appointments, and maintaining follow up care [12]. Respondents in the current study reporting less support regarding medication reminders were more adherent with medication regimens over a 7-day period. While this may seem counterintuitive, there may be other intrinsic motivating factors operating here [13]. For instance, adherence may be related to higher health literacy, knowledge of viral load, goal cognitions, and better overall health [14,15]. Missed dosages occurred when clients from our study forgot, were away from home, and had a change in daily routine. Kim *et al.* found self-motivation (e.g., having a routine) to be most helpful among HIV+ adolescents. However, reminders from friends and family were cited secondary to having a routine which conflicts with our findings [13]; thus, self-motivation provides some context to this surprising finding and warrants further exploration. Furthermore, although the seven-day adherence rates between those who received support/reminders *vs.* those who did not was significant, the difference was not large (43% *vs.* 57%).

Similarly, individuals may have institutional support from a church, without disclosing their HIV status. A subset of our sample reported being very satisfied with support received from his/her church, with a smaller proportion reporting congregation members helped remind him/her to take their medications. These numbers may be the result of having a mostly male sample who traditionally are less likely to be regular church-goers and to have a church home than women; it still remains noteworthy to highlight that in some instances the church may facilitate linkage and retention in care. George *et al.* echoed this point with their findings demonstrating HIV-related organizations, support groups, and churches also provided some general care; but noting this being less so than family and friends [12]. This study also found that while churches were generally considered a source of support, some participants did experience instances of HIV-related stigma, which is congruent with our survey data [12]. Other research suggests communities of faith may perpetuate HIV-related stigma. Findings from Wilson *et al.* explored church ideologies around sexuality and health. Findings demonstrate how church doctrines and attitudes/beliefs among clergy and congregation members may concurrently impede and facilitate the faith community’s response to addressing the needs of sexual minorities [16].

It is imperative to consider and acknowledge the variability of religious denominations in general, and Historically Black Churches specifically, as these institutions are not monolithic. Some churches by nature are more progressive than others regarding HIV/AIDS prevention and treatment, and thus may be more accepting and affirming [17]. Pichon and colleagues found faith leaders were comfortable discussing sensitive topics such as sexual behaviors and sexual communication as they relate to HIV risk with adolescent congregants as part of a faith-based HIV prevention pilot program [18]. However, denominational differences were found across participants. For instance, Baptist faith leaders were significantly more likely than non-Baptist faith leaders to be comfortable discussing homosexuality [18]. Whereas some faith-leaders are directly accountable to a denominational governance structure, Baptist churches are typically governed by their own by-laws and organizational structure, and thus have more flexibility with how they operate. Other research highlights the growth and evolvement of belief systems and practices among faith leaders and religious congregations with regard to HIV prevention and HIV testing 30 years into the epidemic [19,20].

Despite the progress, researchers must remain mindful of HIV-related stigma while engaging churches in these efforts. It would behoove faith and academic partnerships to work toward strengthening inter-denominational ties, providing cultural enrichment training to congregations and church leaders, and actively involving congregations and members of the infected/affected community in the conceptualization of social support initiatives for HIV+ church members. Many churches have active health ministries that could be used to garner support for investigating the types of support needed by congregations [21]. Framing HIV prevention as a health issue not a moral issue is paramount. General health promotion messages centered around topics like medication adherence, medical care, and treatment could be disseminated via worship services, sermons, and church bulletins without outing church members or being specific to any one disease. Further, including HIV as part of the already long list of health issues faced by congregations may help to normalize the disease as simply another chronic condition and possibly minimize associated stigma.

### 4.1. Strengths and Limitations

This was a convenience-based sample of medically compliant clients recruited from local Ryan White medical and support service providers. Thus, this group may differ from those not receiving Ryan White-funded services, those less medically compliant, and those with private insurance. We relied on self-report to assess medication adherence as opposed to using electronic monitors or medical records given external time constraints. We used a global assessment of satisfaction with social support instead of individual network member by network member survey item. We did not assess religious affiliation and level of spirituality.

Several noteworthy strengths are worth mentioning. Survey interviewers included providers familiar with client viral loads and their medication taking behaviors, which may reduce the social pressure/desirability of overstating medication adherence. Secondly, we assessed participant satisfaction with the church in providing support to clients both in general and specifically as it relates to medication adherence reminders. This contributes to the growing body of research to understand the possible role of religious institutions in promoting health-seeking behaviors among HIV clients particularly in the Mid-South. Thirdly, exploring the role of the faith community within the Memphis Part A Program structured needs assessment extends beyond HRSA requirements and adds to our understanding of other avenues to link and retain HIV-positive adults in care.

### 4.2. Research and Practice Implications

While Ryan White is currently the main source for a comprehensive continuum of care and supportive services for PLWH with fewer economic resources, more clients may become eligible for private insurance as changes take place with the Affordable Care Act; however, the impact of these changes is unknown and the utility of alternative sources of support such as family, friends, and the church will need further exploration and consideration. The contextual factors of living with HIV in the Mid-South are equally important given the power of faith and religion. The influence of these factors may contribute to engagement and retention in care. A logical next step includes an assessment of the spiritual needs among our HIV population and how the church could serve as a facilitator to increasing medication and adherence support through addressing those spiritual needs. Other questions are raised: Are people who are more spiritually satisfied/satiated, more likely to be adherent? What are the pathways by which faith-based social support translates to adherence/non-adherence? 

## 5. Conclusions

Given the poor adherence to antiretroviral medications found in this study, consideration of how best to integrate interpersonal supports from friends, family, and the church should be explored in the context of addressing the full continuum of HIV care. Churches are in a unique position to provide spiritual support to families, and could potentially expand the scope of ministry to encourage preventative health screenings, including HIV testing, and to offer support to those newly infected with HIV by assisting with disclosure and linkage to medical care, and encouraging adherence to medical care and treatment. Additional research is imperative to fully understand the social and contextual factors that prevent medication adherence, and faith-based approaches involving friends, family, and clergy in the development of spiritually grounded interventions are warranted to minimize HIV related stigma and to support those infected and affected by HIV.
